# A rare acneiform eruption presentation of Kikuchi-Fujimoto disease presenting with concurrent systemic lupus erythematosus

**DOI:** 10.1016/j.jdcr.2023.08.028

**Published:** 2023-08-31

**Authors:** Morgan E. Decker, MacKenzie Griffith, Aadil Ahmed, Pamela Madu, Penelope K. Skopis

**Affiliations:** a Department of Dermatology, Rush University Medical Center, Chicago, Illinois; b Rush Medical College, Rush University Medical Center, Chicago, Illinois; c Illinois Dermatology Institute, Chicago, Illinois

**Keywords:** acneiform eruption, Kikuchi disease, Kikuchi-Fujimoto disease

## Introduction

Kikuchi-Fujimoto disease (KFD) is a rare benign histiocytic necrotizing lymphadenitis of unknown etiology.^1^ It is a self-limited condition that typically manifests in young adults in the third decade of life and classically presents with a flu-like prodrome followed by fevers and cervical lymphadenopathy.^2^ The underlying cause is unknown, but it has been hypothesized to represent an immune-mediated response to a viral infection.^3^ Cutaneous manifestations associated with KFD vary and may include erythematous macules, patches, papules or plaques, malar erythema, pruritus, or oral ulcers.^3^ Herein, we present a rare case of KFD presenting with acneiform skin eruption in a patient with systemic lupus erythematosus (SLE).

## Case report

A 48-year-old female with a past medical history of Graves disease, hypertension, and anxiety presented to the emergency department with several weeks of nausea, vomiting, and generalized fatigue. Two weeks prior to presentation, the patient developed a nonpruritic nontender rash on the right cheek that subsequently spread to her forehead, left cheek, lips, and nose. The patient denied a history of acne and denied any new hair or skincare products. Review of systems was negative for oral ulcers.

**Fig 1:**
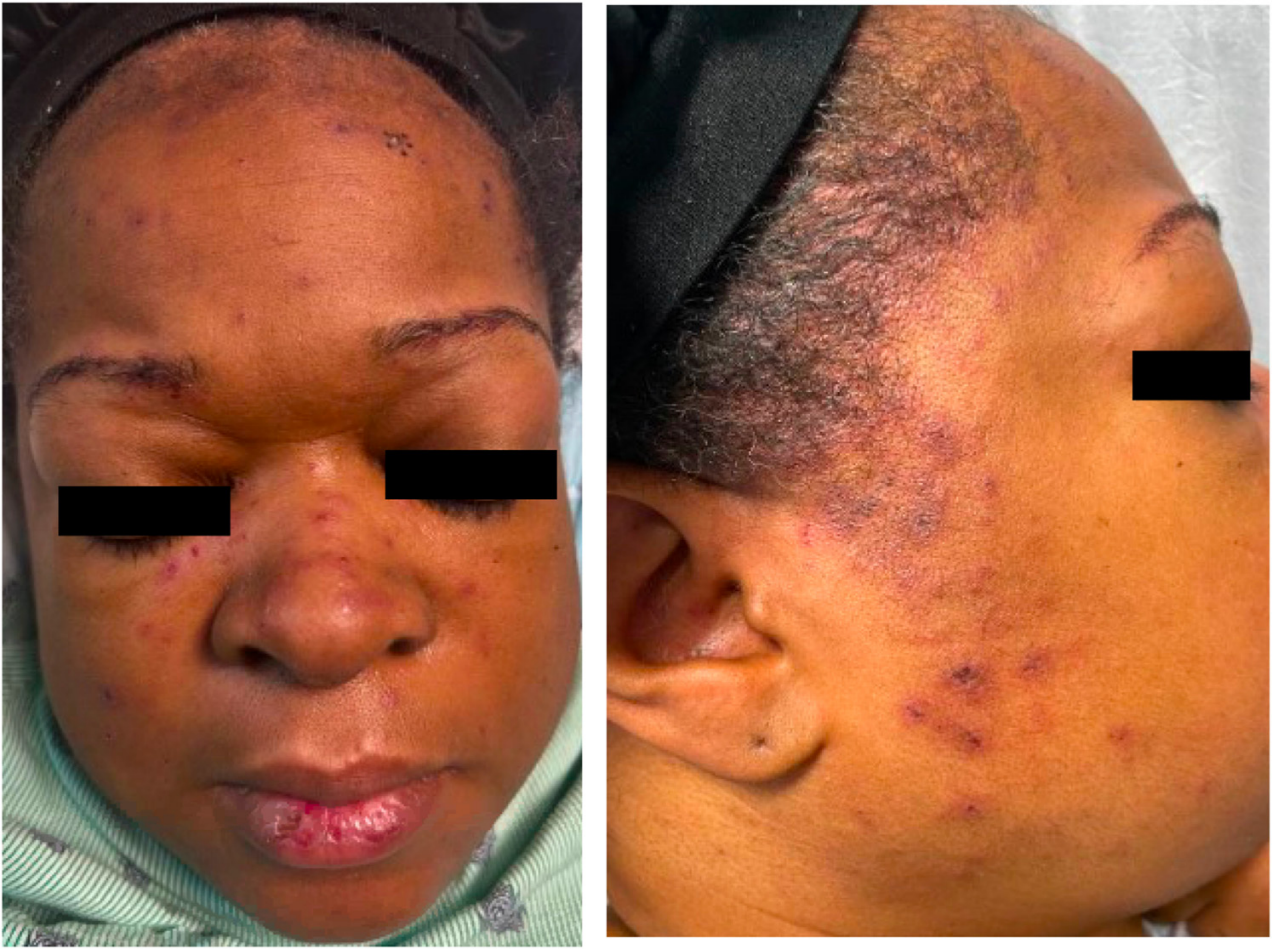
Clinical photographs.

On admission, the patient was noted to have intermittent fevers, axillary lymphadenopathy, and multiple erythematous-to-violaceous follicularly-centered macules and papules with central crust on the preauricular cheeks, nasal dorsum, and forehead at hairline (Fig 1).

**Fig 2:**
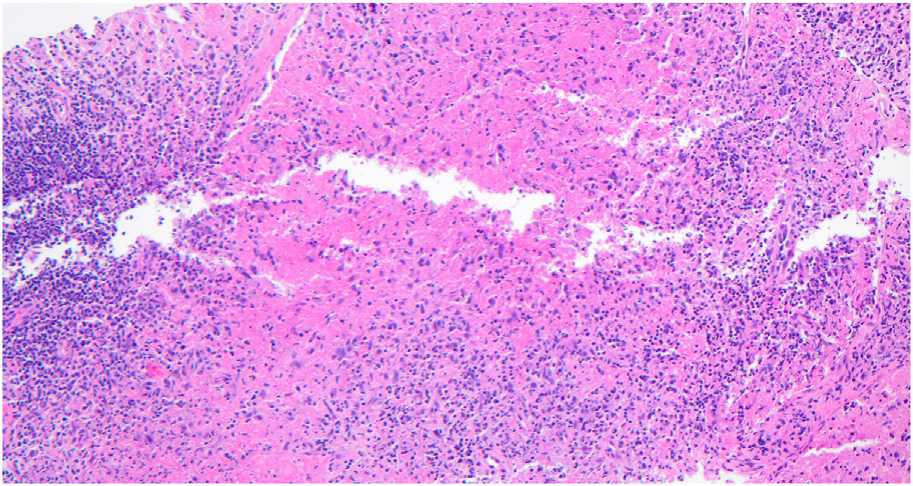
Hematoxylin and eosin stain axillary lymph node biopsy.

**Fig 3:**
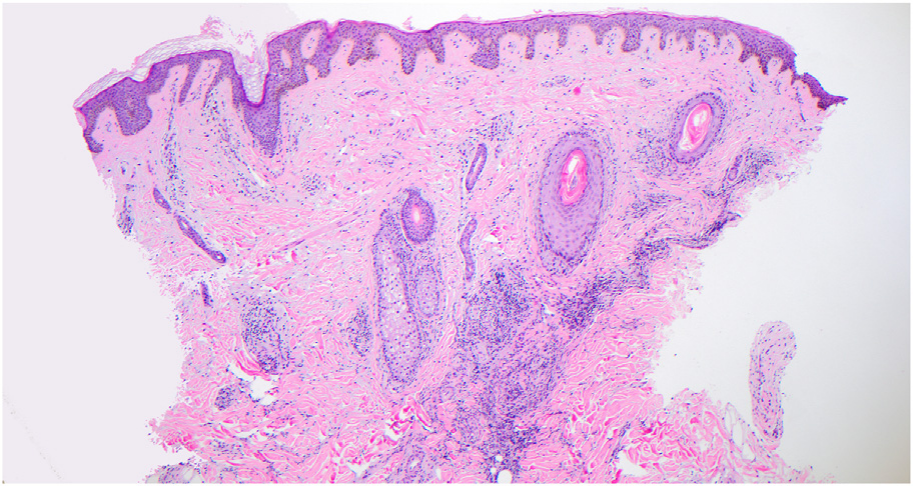
Hematoxylin and eosin stain punch biopsy of lesion.

**Fig 4:**
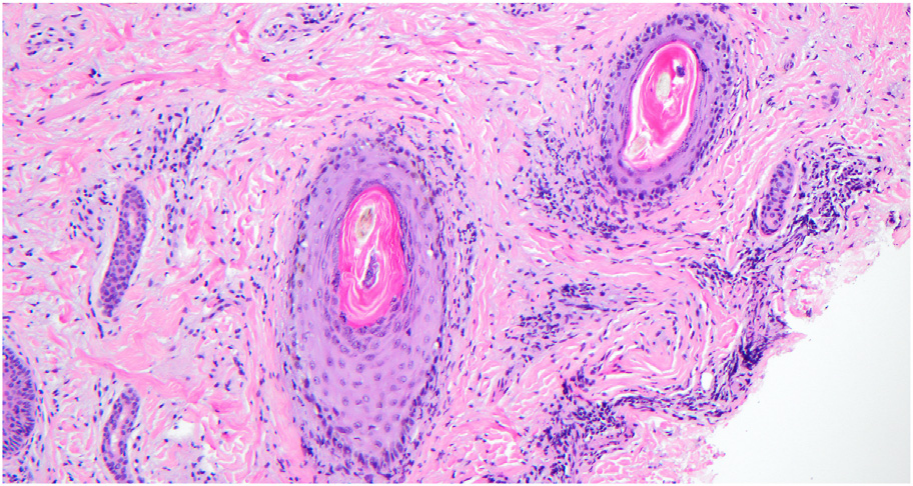
Hematoxylin and eosin stain demonstrating focal vacuolar changes within follicular epithelium.

Laboratory testing revealed leukopenia, low C3 (78), positive antinuclear antibodies (ANA) (titer ≥1:1280), and positive anti-Smith and anti-ribonucleoprotein antibodies. The American College of Rheumatology classification criteria were utilized to diagnose SLE, wherein a patient must have an ANA titer ≥1:80 and total clinical and immunologic score greater than 10 for diagnosis. The patient’s ANA titer (≥1:1280), fever (2), leukopenia (3), and positive anti-Smith antibodies (6) resulted in a score of 11. A core needle biopsy from the right axillary lymph node demonstrated large areas of necrosis with abundant apoptotic material and preserved lymph node architecture (Fig 2). Occasional histiocytoid cells were appreciated in the rims of the necrotic areas. Punch biopsy of a representative lesion on the forehead revealed a superficial-to-mid perivascular and periadnexal infiltrate within the dermis (Fig 3). No vacuolar changes were seen at the dermoepidermal junction; however, focal vacuolar changes were seen along the follicular epithelium with nonneutrophilic karyorrhexis (Fig 4). Grocott-Gomori methenamine silver and Fite stains were negative for fungus and *Mycobacterium leprae*, respectively. Given the constellation of symptomatology, serologies, and histopathology, the patient was diagnosed with KFD with concurrent SLE.

The patient was started on hydroxychloroquine 400 mg daily, prednisone 40 mg daily, and topical tacrolimus 0.1% ointment twice daily for facial rash. At 1-week follow-up, the patient’s acneiform eruption had improved and residual postinflammatory hyperpigmentation was noted.

## Discussion

First described in 1972 in Japan, KFD, also known as necrotizing histiocytic lymphadenitis, is a benign, self-limited condition characterized by fever and lymphadenopathy with nausea, vomiting, weight loss, myalgias, and skin lesions reported in a subset of affected patients.^1^^,^^4^ Skin eruptions have been reported in 40% of KFD cases. The most common skin finding in KFD is a nonspecific rash, manifesting as erythematous macules or patches, papules or plaques, or a maculopapular eruption.^3^ Additional reported cutaneous findings include subcutaneous nodules and erythema multiforme.^5^

Although the underlying cause of KFD is unknown, it has been hypothesized to represent an exuberant CD8^+^ T cell-mediated immune response to an infectious agent in genetically susceptible individuals.^6^ Epstein-Barr virus, HIV, human herpes virus 6, human herpes virus 8, and human T-lymphotropic virus have all been suggested as possible inciting agents for the disease.^1^^,^^7^ KFD typically affects young adults less than 40 years of age and has been associated with multiple autoimmune conditions, including Graves disease, Sjogren syndrome, rheumatoid arthritis, Still disease, and SLE.^1^

While the relationship between KFD and SLE is not completely understood, the association between the 2 conditions has been reported.^2^ SLE can present before, after, or, as was the case for our patient, at the same time as KFD.^8^ Within cases in the literature describing associated KFD and SLE, 51% of cases described the 2 conditions presenting simultaneously, 31% of cases reported a SLE diagnosis following KFD, and 18% described patients with SLE prior to KFD.^9^ Cutaneous lesions have been reported in 80% of SLE-KFD cases.^9^ Cutaneous findings reported in previous SLE-KFD cases include malar erythema (37.5%), oral aphthae (26.7%), generalized rash (11.5%), discoid lupus (10.6%), cutaneous vasculitis (8%), interface dermatitis (3.4%), and cutaneous nodes (2.7%).^9^ Interestingly, among KFD cases presenting with acneiform eruptions, concurrent SLE was not reported.^3^^,^^10^

Definitive diagnosis requires lymph node biopsy, and histopathology can be important in distinguishing KFD from other more serious lymphadenopathies including infectious lymphadenopathies (eg, toxoplasmosis, mononucleosis, tuberculosis), lymphoma, and metastatic disease.^1^^,^^2^ KFD often resolves spontaneously within months of diagnosis; however, in severe cases nonsteroidal antiinflammatory agents, oral corticosteroids, hydroxychloroquine, methotrexate, and intravenous immunoglobulin have been used.^5^

In conclusion, KFD should be considered in a patient presenting with fevers, lymphadenopathy, and nonspecific cutaneous manifestations for which another cause cannot be identified. A lymph node biopsy is essential to establish the diagnosis of KFD, and given the association between SLE and KFD and variation in which condition presents first, ANA screening with serial clinical reevaluation for the development of autoimmune disease, particularly SLE, is recommended when treating this condition.

## Conflicts of interest

None disclosed.
